# Recent Advances of Transition Metal Basic Salts for Electrocatalytic Oxygen Evolution Reaction and Overall Water Electrolysis

**DOI:** 10.1007/s40820-023-01038-0

**Published:** 2023-03-02

**Authors:** Bingrong Guo, Yani Ding, Haohao Huo, Xinxin Wen, Xiaoqian Ren, Ping Xu, Siwei Li

**Affiliations:** 1 Institute of Industrial Catalysis, School of Chemical Engineering and Technology, Xi’an Jiaotong University, Xi’an, 710049 People’s Republic of China; 2 Institute of Carbon Neutral Energy Technology, School of Energy Science and Engineering, Harbin Institute of Technology, Harbin, 150001 People’s Republic of China; 3 MIIT Key Laboratory of Critical Materials Technology for New Energy Conversion and Storage, School of Chemistry and Chemical Engineering, Harbin Institute of Technology, Harbin, 150001 People’s Republic of China

**Keywords:** Transition metal basic salts, Electrocatalytic, Oxygen evolution reaction (OER), Overall water electrolysis

## Abstract

Electrocatalytic oxygen evolution reaction (OER) has been recognized as the bottleneck of overall water splitting, which is a promising approach for sustainable production of H_2_. Transition metal (TM) hydroxides are the most conventional and classical non-noble metal-based electrocatalysts for OER, while TM basic salts [M^2+^(OH)_2-x_(A^m−^)_x/m_, A = CO_3_^2−^, NO_3_^−^, F^−^, Cl^−^] consisting of OH^−^ and another anion have drawn extensive research interest due to its higher catalytic activity in the past decade. In this review, we summarize the recent advances of TM basic salts and their application in OER and further overall water splitting. We categorize TM basic salt-based OER pre-catalysts into four types (CO_3_^2−^, NO_3_^−^, F^−^, Cl^−^) according to the anion, which is a key factor for their outstanding performance towards OER. We highlight experimental and theoretical methods for understanding the structure evolution during OER and the effect of anion on catalytic performance. To develop bifunctional TM basic salts as catalyst for the practical electrolysis application, we also review the present strategies for enhancing its hydrogen evolution reaction activity and thereby improving its overall water splitting performance. Finally, we conclude this review with a summary and perspective about the remaining challenges and future opportunities of TM basic salts as catalysts for water electrolysis.

## Highlights

- We summarize the recent advances of transition metal basic salts and their application in oxygen evolution reaction (OER) and further overall water splitting.
- The structure evolution of transition metal basic salts during OER and the impact of F^−^, Cl^−^, CO_3_^2−^ and NO^3−^ on the OER performance are highlighted

## Graphical abstract

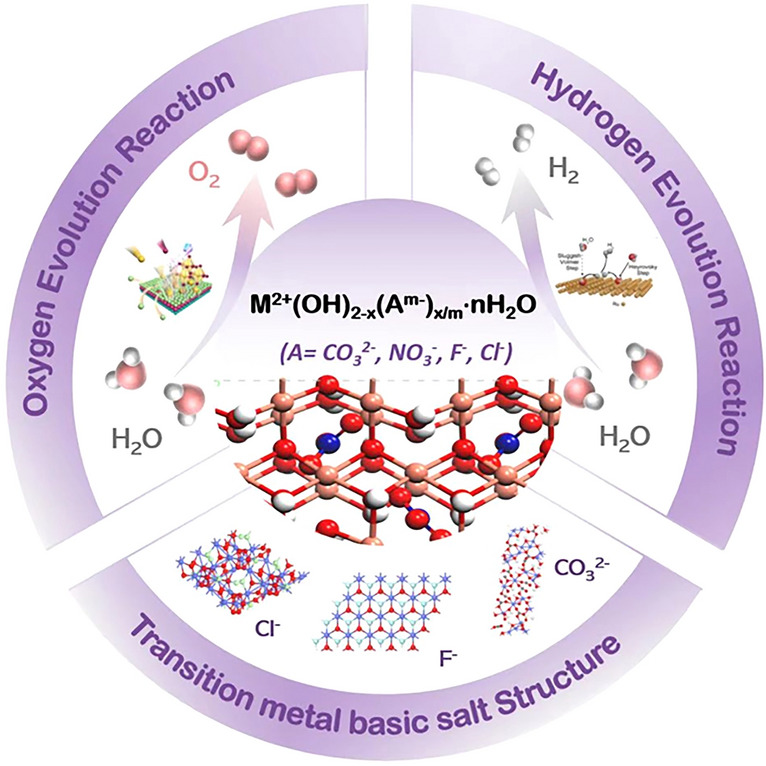

## Introduction

Along with the intensification of industrial production and social activities, the demand of human society for energy has never been so large as today [1–5]. However, the primary source of energy supply continues to rely on the unrenewable fossil fuels that are pretty scarce in reserves [6–11]. Thus, the energy issues caused by the depletion of fossil fuels certainly will give a rise to the huge demand for the clean energy [12–17]. Hydrogen, as a green and efficient energy, has been considered as a clean energy resource to deal with this potential crisis [18–23]. Electrocatalytic water splitting into hydrogen and oxygen provides a feasible pathway to produce clean energy resources [24–28], which contains two half-reactions, hydrogen evolution reaction (HER) and oxygen evolution reaction (OER) [29–32]. The anodic OER has been considered as the bottle neck for water splitting due to the sluggish four-electron transfer process and the formation of O–O bond [33–39]. Noble metal oxides (RuO_2_ and IrO_2_) are the benchmark catalysts for OER [40–45]. However, their large-scale application are hindered by the high cost and relatively low long-term stability [46–53]. Therefore, it is of great importance to develop non-noble metal-based electrocatalysts towards OER [54–60].

Till now, plenty of non-noble metal-based materials such as oxides [61–64], (oxy)hydroxides [65–69], sulfides [70–72], selenides [73–75], phosphides [76–79], phosphates [80–82] have been developed as efficient electrocatalysts towards OER. Among the reported electrocatalysts, transition metal hydroxides are the most conventional electrocatalysts, because they are easily prepared but exhibit high activity [83–87]. TM basic salts can be normally formulated as M^2+^(OH)_2-x_(A^m−^)_x/m_ · nH_2_O (A = CO_3_^2−^, NO_3_^−^, F^−^, Cl^−^, etc.), which has another anion compared to TM hydroxides. Moreover, TM basic salts possess abundant base active sites [88], unique channel structure [89], and special electronic configuration [90], which are beneficial to OER performance. As a result, since the pioneering work in 2014, TM basic salts with CO_3_^2−^, NO_3_^−^, F^−^ or Cl^−^ have been reported to exhibit better OER performance than their hydroxide counterparts and among the best non-noble metal-based electrocatalysts regardless of the type of anion [91–94]. Furthermore, highly active TM basic salt-based catalysts have been designed for OER and even overall water splitting through cation doping and construction of heterostructure [95–98]. Of note is that TM basic salts are partially or even fully oxidized to the MOOH during OER process. This makes the anion effect on OER performance, the most basic scientific question in this area, complex and controversial. In brief, it is no doubt that TM basic salts are one of the most promising pre-catalysts for OER, however, there has not been a comprehensive review for TM basic salts except for two reviews on TM carbonate hydroxides [29, 99].

**Fig. 1:**
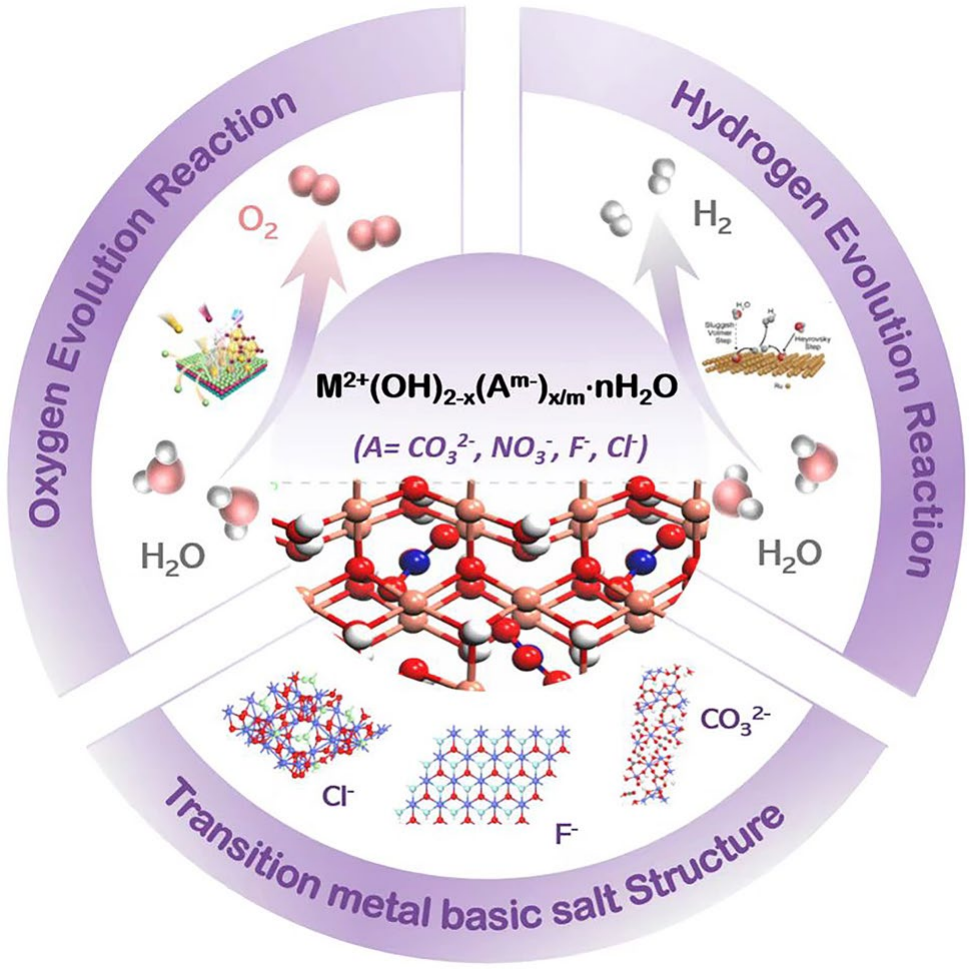
Transition metal basic salts for OER and overall water splitting

In this review, we summarize the great progresses have been made for TM basic salt-based electrocatalysts towards OER and further overall water splitting (Fig. 1). First, we provide a brief introduction to the structure of transition metal basic salts. Then, we categorize TM basic salt-based OER pre-catalysts into 4 parts according to the kind of anions (i.e., F^−^, Cl^−^, CO_3_^2−^ and NO_3_^−^). Besides for the introduction of these pre-catalysts, we focus on discussing the effect of anion on the OER performance. Of note is that we restrict of discussion to those works that have taken the in-situ oxidation of TM basic salts in this section. Afterwards, we introduce the design idea (e.g., metal doping, construction of heterostructure) for construction of TM basic salt-based catalysts to enhance their HER performance, thus improving the overall water splitting. At last, we discuss the challenges and opportunities for this kind of emerging catalysts.

## Structure of Transition Metal Basic Salts

Transition metal basic salts are defined as salt compounds composed of transition metal cations and hydroxide anions (OH^−^), in which part of OH^−^ are replaced by other anions formed from the strong monoprotic acids or the weak diprotic acid. Thus, transition metal basic salts show very poor tolarance to acid solution, and can only be used in alkaline and neutral conditions. In general, it can be normally formulated as M^2+^(OH)_2-x_(A^m−^)_x/m_ · nH_2_O [100–102], where the M^2+^ represents the transition metal cations (Fe^2+^, Co^2+^, Ni^2+^, etc.), and the A^m−^ stands for the anions of acids, such as CO_3_^2−^, NO_3_^−^, F^−^, Cl^−^, SO_4_^2−^, SeO_4_^2−^ and CH_3_COO^−^ [103–109]. We mainly focus on the four type of metal basic salts (CO_3_^2−^, NO_3_^−^, F^−^, Cl^−^) that have been developed as pre-catalysts for OER.

**Fig. 2:**
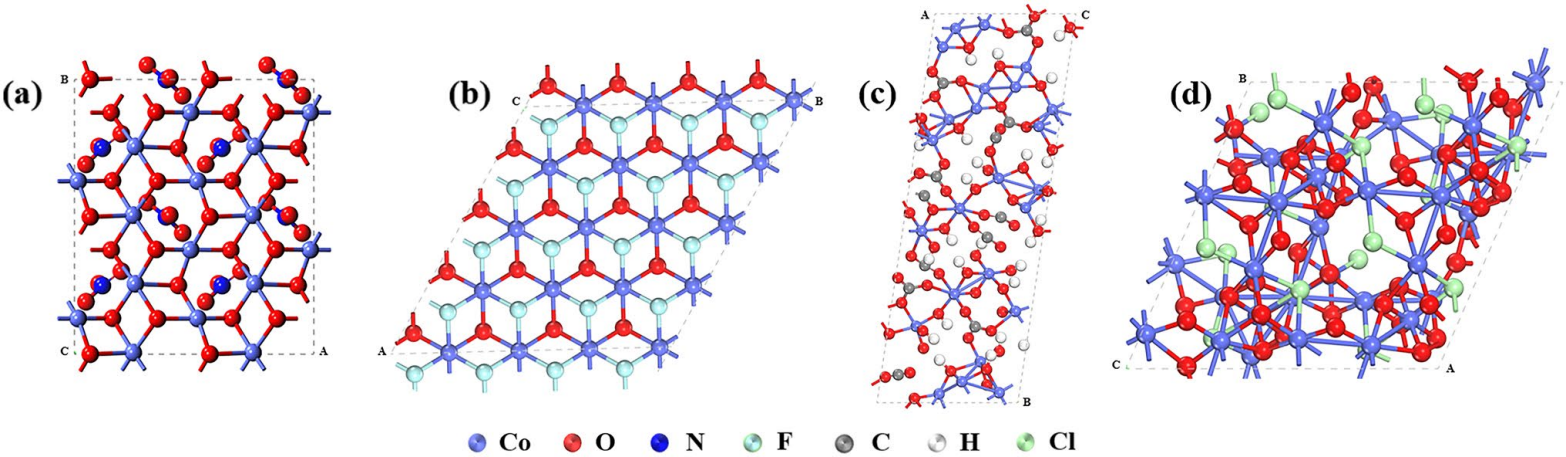
Crystal structure of **a** Co_2_(OH)_3_NO_3_, **b** Co(OH)F, **c** Co_2_(OH)_2_CO_3_, and **d** Co_2_(OH)_3_Cl

Most transition metal basic salts, but not all of them, are considered to have layered structure. The structures of transition metal basic salts containing ions of NO_3_^−^, Cl^−^, CO_3_^2−^, or F^−^ are described detailed below. (1) Transition metal basic salt consisting of NO^3−^ has a formula of M_2_(OH)_3_NO_3_, which is also known as transition metal nitrate hydroxides (TMNHs) [110]. This type of basic salts have two distinct structures [111]. For the single layered M_2_(OH)_3_NO_3_, its structure consists of brucite-like M(OH)_2_ layers, where 1/4 of oxygen atoms belong to nitrate groups [112] and the octahedral sites are singly occupied by the divalent metal ions (Fig. 2a). The structure of the double-layered M_7_(OH)_12_(NO_3_)_2_ has not been reported. Divalent metal ions are assumed to be coordinated with octahedral and tetrahedral oxygen atoms in the postulated structure. (2) Transition metal fluoride hydroxides (TMFHs) have a formula of M(OH)F, which show similar electronic structure and the electrochemical properties with metal hydroxides-based semiconductors due to its inherent structure (Fig. 2b) [105]. (3) TM carbonate hydroxides (TMCHs) are a kind of layered hydroxide salts, consisting of positively charged cations and intercalated anions in the interlayer region [113, 114]. In transition metal carbonate hydroxides, the M ions coordinate with two oxygen atoms from CO_3_^2−^ and four OH^−^ ions to form distorted octahedron with (4 + 2) coordination (Fig. 2c) [115]. (4) Transition metal oxychlorides (such as Co_2_(OH)_3_Cl) are known as TMClHs due to the existence of Cl^−^. It presents a pyrochlore-like hexagonal structure with Co^2+^ occupying its octahedral sites (Fig. 2d) [116, 117]. Among the above basic salts, carbonate and nitrate hydroxides possess typical layered structure, which not only assists the mass transfer and ions migration between the electrode and electrolyte [29], but also provides large surface area that generates ample accessible active sites toward OER [118]. Besides, the above-mentioned transition metal basic salts can be prepared at facile conditions. For instance, the transition metal basic salts with CO_3_^2−^, NO_3_^−^, F^−^, Cl^−^ can be prepared via typical solvothermal method at 90 ~ 180 °C [105, 119–121], in which the type of anion can be controlled by using corresponding precursor or inducing agent [119, 122]. Of note is that MNH can also be obtained through molten salt decomposition of corresponding MNO_3_ at 85 ~ 140 °C [123]. Thus, it is of great importance to develop transition metal basic salts as pre-catalyst for OER.

## Transition Metal Basic Salts for OER

This section is further divided into four parts according to the kind of anion, because it is generally acknowledged that anion type is regarded to determine the property and catalytic performance of transition metal basic salts. In this section, the effect of anion on the OER performance is a key point. Of note is that transition metal basic salts undergo partially or even fully oxidation during OER, so we mainly discuss the views about the anion effect which take the structure evolution into consideration.

### Cl^−^

**Fig. 3:**
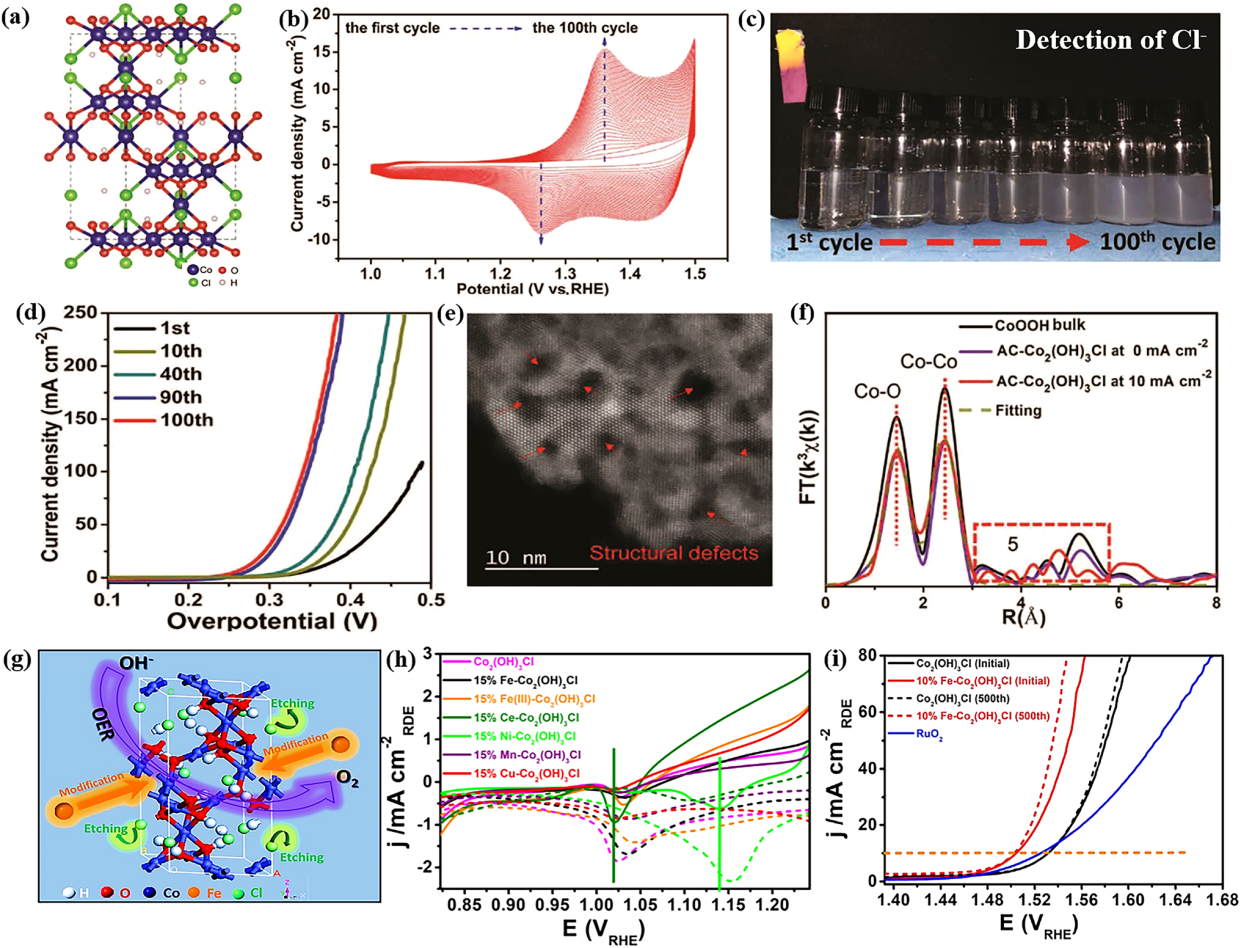
**a** Crystal structure of Co_2_(OH)_3_Cl. **b** CV curves of 100 cycles in 1.0 M KOH with a scan rate of 100 mV s^−1^. **c** Optical photos for the detection of Cl^−^ in electrolyte with the increase of the CV cycles. **d** LSV curves with the increase of the CV cycles. Scan rate: 5 mV s^−1^. **e** STEM image of the AC-Co_2_(OH)_3_Cl. **f** Fourier-transformed Co K-edge EXAFS spectra and corresponding fitting curves for the activation derived-Co_2_(OH)_3_Cl. Reproduced with permission from Ref. [124]. Copyright 2019, Wiley–VCH Verlag GmbH & Co. KGaA, Weinheim. **g** Schematic illustration of the Fe modification to Co_2_(OH)_3_Cl. **h** LSV curves of oxygen reduction reaction before (solid line) or after 500 CV cycles (dashed line) in O_2_-saturated 1 M KOH (negative scan). **i** LSV curves of the Fe modified Co_2_(OH)_3_Cl. Reproduced with permission from Ref. [125]. Copyright 2020, Elsevier Inc

Song et al. first developed Co_2_(OH)_3_Cl as a pre-catalyst for OER (Fig. 3a), and further carried out a comprehensive study for the activation and structure evolution process during OER [124]. The authors discovered that Co(II) in Co_2_(OH)_3_Cl was converted to Co(III) at the first 100 cyclic voltammetry (CV) cycles (Fig. 3b), and Cl^−^ was dissolved into the electrolyte (Fig. 3c). Simultaneously, the OER performance improved continuously during the first 100 CV cycles (Fig. 3d), indicating that the activation of Co_2_(OH)_3_Cl was originated from plenty of structural defects (Fig. 3e). In fact, Co_2_(OH)_3_Cl was found to fully oxidized to CoOOH after the OER process. An important contribution of this work is that the authors employed a series of *operando* spectroscopic methods such as X-ray absorption spectroscopy (XAFS) to directly show the structure evolution of Co_2_(OH)_3_Cl during OER (Fig. 3f). As a result, the coordinatively unsaturated Co sites resulted from the Cl^−^ leaching were demonstrated to be responsible for the outstanding OER performance. In our view, Song et al. not only provide a self-consistent explanation for the anion effect of transition metal basic salt-based OER pre-catalysts, but also contribute to the methodology for understanding the activation mechanism for other kinds of OER pre-catalysts.

Regulating surface states and electronic structures of transition metal basic salts through elemental doping has been believed as a valid strategy to improve its OER activity. For example, Hong and his co-workers further enhanced the OER activity of Co_2_(OH)_3_Cl through the modification of small amount Fe elements (Fig. 3g) [125]. By comparing the integral areas of O_2_ reduction reaction peaks in LSV curves (Fig. 3h), the author demonstrated that doping Fe into the Co_2_(OH)_3_Cl lattices with moderate amount facilitated the etching of surface lattice Cl^−^ and created more surface vacancies to absorb oxygen species. Besides, the modification of Fe led to a moderate surface oxygen adsorption affinity, which is favorable to activate the intermediate oxygen species. These merits made the activated sample show good quality OER activity, achieving a low overpotential of 273 mV at 10 mA cm^−2^ (Fig. 3i).

### F^−^

**Fig. 4:**
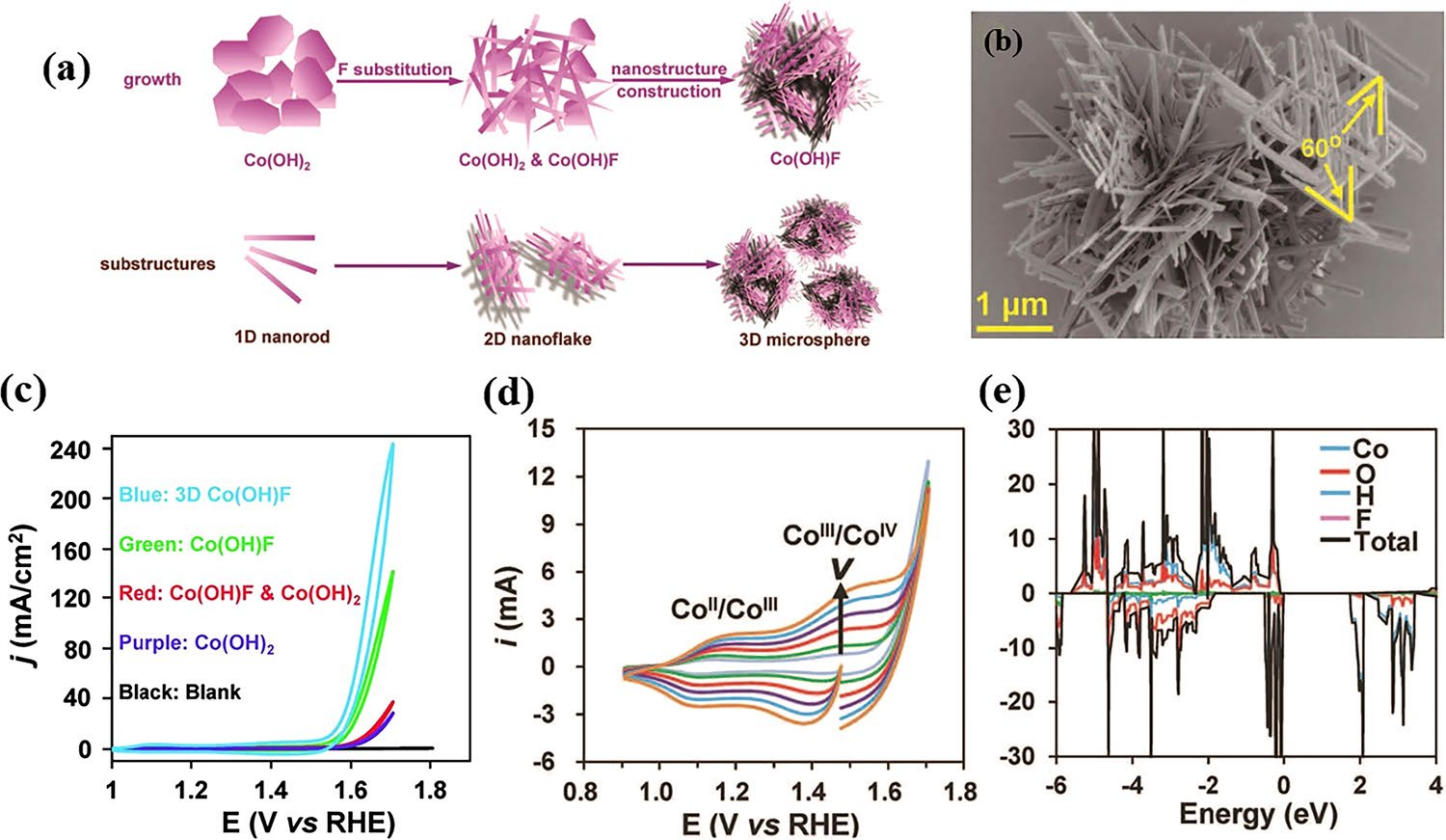
**a** Schematic of the growth and substructures, **b** SEM image of the hierarchical 3D Co(OH)F microspheres. **c** CVs of the Co-based pre-catalysts. **d** CV current–potential responses of the Co(OH)F sample at different scan rates: 0.5 V s^−1^ (azure), 1 V s^−1^ (green), 2 V s^−1^ (red), 3 V s^−1^(purple), 4 V s^−1^ (blue), and 5 V s^−1^ (brick red). **e** Calculated densities of states of Co(OH)F. Reproduced with permission from Ref. [105]. Copyright 2017, Wiley–VCH Verlag GmbH & Co. KGaA, Weinheim

The pioneering work for Co(OH)F-based OER pre-catalyst was reported by Cao and his co-workers [105]. As shown in Fig. 4a, b, the authors developed an elegant synthetic strategy to fabricate hierarchical 3D Co(OH)F microspheres with 2D nanoflakes woven by single-crystal 1D nanorods. This unique superstructure endowed the Co(OH)F pre-catalyst with the virtues of all dimensions (1D, 2D and 3D), including facilitated mass and charge transfer and enlarged electrochemical active surface area. This led to remarkable OER activity of the 3D Co(OH)F catalyst, requiring an overpotential of 313 mV to deliver a current density of 10 mA cm^−2^ (Fig. 4c), which was significantly lower than other kinds of Co(OH)F pre-catalyst as well as Co(OH)_2_. Impressively, the authors noticed the structure evolution of Co(OH)F during OER and provided a self-consistent explanation for the effect of F^−^, even if this was one of the earliest works in this field. Specifically, the author discovered that the surface of Co(OH)F was oxidized to Co^III^/Co^IV^ through CV test and XPS (Fig. 4d), while the bulk phase kept unchanged, which was different from the behavior of Co_2_(OH)_3_Cl. The authors used a core–shell model to explain the effect of F^−^ on the different OER performance between Co(OH)F and Co(OH)_2_. The theoretical calculations indicated that the F^−^ ions in Co(OH)F led to higher conductivity and charge mobility than those of Co(OH)_2_ (Fig. 4e). It should also be pointed out that other factors such as the adsorption of F-, surface defects and different valence state of Co cannot be excluded. Nevertheless, it is no doubt that the synthetic strategy and the reasonable core–shell explanation of this work paves a way for TM basic salt-based OER pre-catalysts. After this pioneering work, Cao’s group further investigated the impact of phase transition on Co(OH)F, and employed Co(OH)F as template to synthesize CoO and CoP for OER [126, 127].

**Fig. 5:**
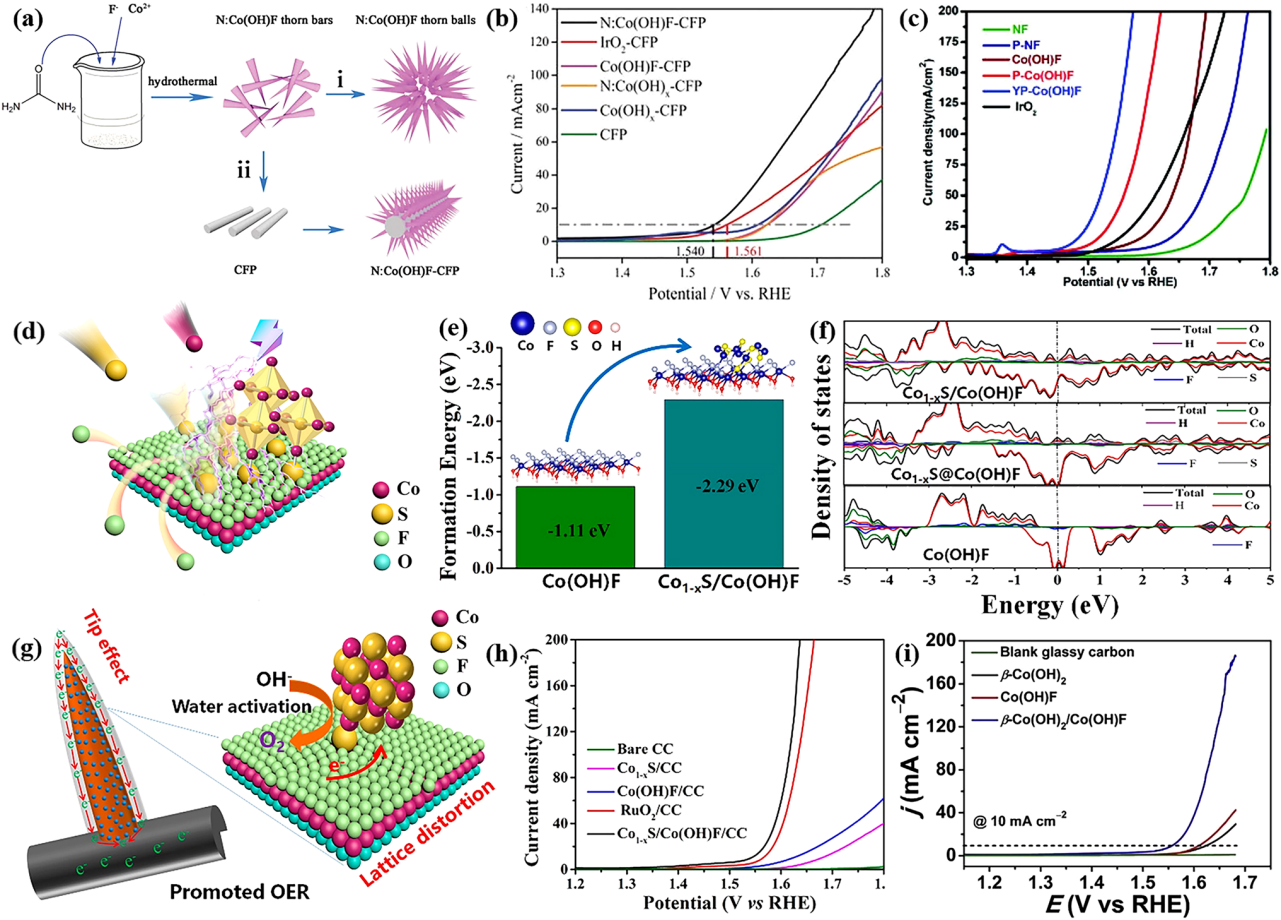
**a** Schematic representation of the growth process of the 3D needlelike N:Co(OH)F array structure on CFP. **b** LSV curves of the N:Co(OH)F and series comparison samples. Reproduced with permission from Ref. [130]. Copyright 2018, Royal Society of Chemistry. **c** LSV curves of Co(OH)F, P-Co(OH)F, YP-Co(OH)F, and IrO_2_ for OER in 1 M KOH. Reproduced with permission from Ref. [131]. Copyright 2019, Wiley–VCH Verlag GmbH & Co. KGaA, Weinheim. **d** Schematic diagram of the sulfur atom replacing the fluorine atom. **e** The formation energy of Co_1−x_S/Co(OH)F and Co(OH)F from DFT calculation. **f** DOS curves of Co(OH)F/CC and Co_1−x_S/Co(OH)F/CC. **g** Schematic of Co_1−x_S/Co(OH)F sample improving the OER activity. **h** OER LSV curves of Co_1−x_S/Co(OH)F sample with a scan rate of 20 mV s^−1^ and 80% iR correction. Reproduced with permission from Ref. [132]. Copyright 2022, American Chemical Society. **i** LSV curves of the as-prepared β-Co(OH)_2_, Co(OH)F, and β-Co(OH)_2_/Co(OH)F hybrid. Reproduced with permission from Ref. [133]. Copyright 2018, Elsevier Ltd

Both cation and anion doping methods are used to improve the OER activity of Co(OH)F [128, 129]. For example, Li and co-workers prepared ultralong needle-like N-doped Co(OH)F/carbon fiber paper (CFP) for OER through one-step hydrothermal method (Fig. 5a) [130]. The incorporation of N resulted in more oxygen vacancies in Co(OH)F and thus obtained higher activity for OER (Fig. 5b). Yang and his co-workers reported Co(OH)F nanoarray with P and Y co-doping and supported on nickel foam (NF) for alkaline OER [131]. The doping of P and Y led to higher electron density of Co species. As a result, only a low overpotential of 238 mV required by YP-Co(OH)F electrode to reach an OER current density of 10 mA cm^−2^ (Fig. 5c). The OER performance of these works are very appealing, but it is a pity that the investigation of structure-performance relationship based on fresh pre-catalysts rather than used electrocatalysts or even in-situ observation.

Construction of heterostructure is another conventional strategy for enhancing the OER performance of transition metal basic salts. Very recently, Wang et al. fabricated needle-like Co_1-x_S/Co(OH)F heterostructure on carbon fiber cloth (CC) for OER through atomic substitution strategy (Fig. 5d) [132]. The easy replacement of F by S atoms caused lattice distortion to increase active sites, as well as optimized the adsorption (OH^−^) and desorption (O_2_) energy during OER (Fig. 5e). Meanwhile, due to the tip-enhanced local electric field effect from needle-like structure and favorable electron transfer (Fig. 5f, g), the Co_1−x_S/Co(OH)F/CC catalyst showed good OER activity of 269 mV to deliver 10 mA cm^−2^ (Fig. 5h). Besides, Cao and his co-workers gave a β-Co(OH)_2_/Co(OH)F hybrid by making Co(OH)F nanorods grow along β-Co(OH)_2_ hexagon edges as lateral branches [133]. Due to the unusual epitaxial growth and the efficient mass/charge diffusion, the obtained catalyst of β-Co(OH)_2_/Co(OH)F exhibits high-quality reaction activity with a low overpotential of 329 mV to deliver an OER current density of 10 mA cm^−2^ (Fig. 5i). In addition to as OER active phase, TM basic salts can be used to stabilize noble metal OER catalysts. For instance, Cho and co-workers used 3D hierarchical Co(OH)F nanosheet arrays as support for Ru single atom catalyst [134]. The Ru SAs/Co(OH)F only needed a low overpotential of 200 mV to drive a current density of 10 mA cm^−2^. The author claimed that the robust electronic coupling between Ru single atom and Co(OH)F prevented the oxidation of Ru to RuO_4_^2−^, leading to outstanding activity and durability.

### CO_3_^−^

As mentioned above, TMCHs are a kind of hydroxide salts with layered structure. Such unique structure makes the materials have abundant redox properties and show high accessibility to electrolyte [118], which provides a favorable opportunity for highly efficient electrocatalytic oxygen evolution. In fact, TMCHs-based OER pre-catalysts are the most conventional ones among all the TM basic salts.

The pioneer work of TMCHs-based OER pre-catalysts was performed by Lin and her co-workers at 2014 [91]. 3D hierarchical CoCH with favorable hollow urchin-like structure was fabricated on FTO through hydrothermal method. The catalytic performance is not good enough by current standard (466 mV @ 10 mA cm^−2^), but this work paves the way for a new kind of OER pre-catalyst. Up to now, a great number of works have been reported for TMCHs as pre-catalysts for OER [135–141], and the oxidation of TMCHs during OER process are demonstrated solidly, too. However, it is surprised to see there isn’t any work that discusses the effect of CO_3_^2−^ on the OER performance, which takes the structure evolution of TMCHs during OER process into consideration. Therefore, we think there is room for an important work in this area.

**Fig. 6:**
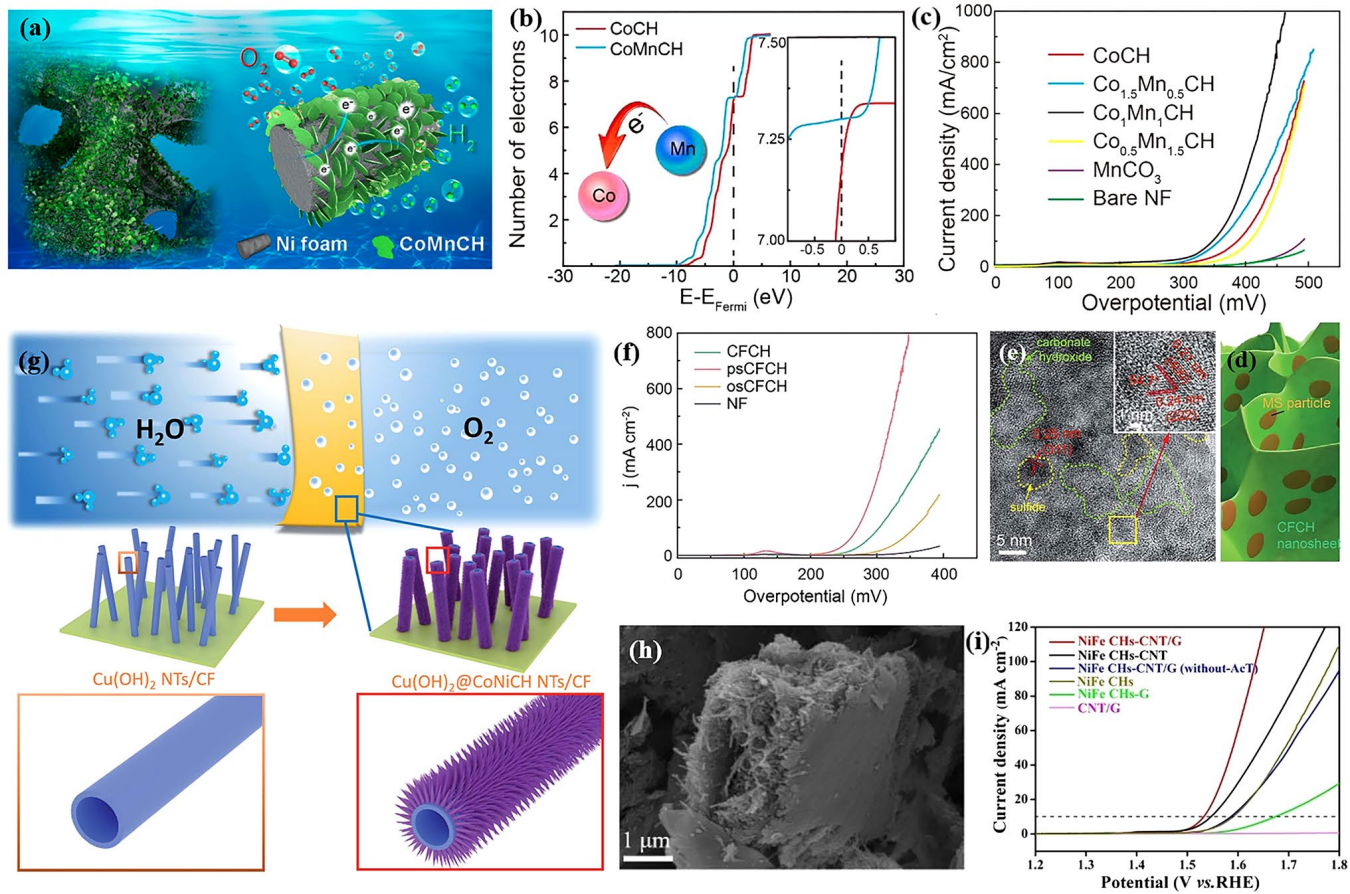
**a** Schematic illustration of Co_x_Mn_y_CH pre-catalyst. **b** The number of electrons in the 3d orbital per Co atom in CoCH and CoMnCH. **c** OER polarization curves of Co_x_Mn_y_CH samples. Reproduced with permission from Ref. [96]. Copyright 2017, American Chemical Society. **d** Schematic illustration of psCoFeCH. **e** HRTEM image (inset: enlarged HRTEM image) of psCoFeCH. **f** OER LSV curves of CoFeCH, psCoFeCH, osCoFeCH, and the NF substrate. Reproduced with permission from Ref. [150]. Copyright 2019, Royal Society of Chemistry. **g** Schematic illustration of the hierarchical Cu(OH)_2_@CoNiCH core/shell NTs grown on CF. Reproduced with permission from Ref. [98]. Copyright 2018, Royal Society of Chemistry. **h** SEM image of NiFeCHs-CNT/G. **i** Polarization curves of NiFeCHs-CNT/G and related samples. Reproduced with permission from Ref. [152]. Copyright 2022, Wiley–VCH GmbH

As the most conventional TM basic salts in the field of OER, different kinds of cations have been used as dopant for TMCHs to enhance its activity [35, 78, 142]. Here, we take CoCH as an example. The most important work for metal-doped CoCHs was carried out by Hu and his co-workers at 2017. They introduced Mn elements into CoCH to boost its electrocatalytic reaction activity through the dual modification of electronic structure and morphology (Fig. 6a) [96]. It has been shown that Mn doping can regulate the nanosheet morphology of CoCH to expose more accessible active sites, as well as make the electron transfer from Mn to Co and thus tune the electronic structure (Fig. 6b). As a result, the optimal CoMnCH/nickel foam (NF) electrode material displayed an unprecedented electrocatalytic OER activity, only requiring a pretty low overpotential of 294 mV to drive a current density of 30 mA cm^−2^ (Fig. 6c). Besides, CoMnCH/NF was also developed as catalyst for overall water splitting for the first time in this work. In our view, the work by Hu’s group is a milestone in this area, which strongly arouses the researchers’ interest in TM basic salts-based OER pre-catalysts. Afterwards, plenty of transition metal elements such as Fe [118, 143], Ni [144–146], W [147], Cu [91] and Cr [148] have been doped into CoCH. Very recently, CoCH with dual dopant such as NFe [149] has also been reported to get improved OER activity. We summarize the OER performance of these works in Table 1. However, the present works usually report one or two kinds of doping element, so there is some variation in the condition of electrochemical test and the property of pure CoCH. Therefore, the question about the best dopant should be systematically investigated by one group, and the structure evolution of CoCH during OER should be considered for the explanation for the trend of performance.

**Table 1:** OER performance of CoCH before and after doping with different cations

Pre-catalysts	Support	Loading amount (mg)	η_10_ (mV) (undoped)	η_10_ (mV)	References
Cu-CoCH	FTO	12 ~ 16	466	606	[91]
Mn-CoCH	NF	/	337 at 30	294 at 30	[96]
Mn-CoCH	GP^a^	/	376	276	[145]
Fe-CoCH	NF	/	280	200	[143]
Fe-CoCH	NF	/	> 270	228	[118]
Ni-CoCH	CC	0.75	/	238	[144]
Ni-CoCH	GP	/	376	266	[145]
Ni-CoCH	GS^b^	0.73	339	141	[146]
W-CoCH	CC	/	332	318	[147]
Cr-CoCH	NF	/	273	203	[148]
NFe-CoCH	CC	2.24	284	235	[149]

^a^Graphite paper

^b^Graphite sheet

The heterostructures of TMCHs and another material such as metal oxides, (oxy)hydroxides, oxysalts, sulfides, carbon nanomaterials and even noble metal nanoparticles have been reported as pre-catalysts for OER. Bimetallic TMCHs are also used to obtain outstanding OER performance. After reporting the CoMnCH system, Wan and Hu’s group further constructed a CoFe carbonate hydroxide and metal sulfide heterostructure (MS/MCH) through partial sulfurization of MCH (Fig. 6d, e) [150]. The author demonstrated that the unique “nanoparticle-in-nanosheet” structure made the electron transfer from MS to CoFeCH, leading to enhanced intrinsic activity (226 mV@10 mA cm^−2^) and durability for OER (Fig. 6f). Another point of interesting is that this electrode material can also reach an industrial current density of 1,000 mA cm^−2^ at a low overpotential of 367 mV, which is crucial for industrial application. Sun et al. developed core–shell hierarchical Cu(OH)_2_@CoNiCH/CF pre-catalyst for OER (Fig. 6g) [98]. Although Co(OH)_2_ itself isn’t an active material for OER, the faster electron transport at interface and easier accessibility of water made the electrode show excellent activity with 288 mV to drive an OER current density of 10 mA cm^−2^. After this work, Sun’s group further interspersed Pd on it to enhance the OER performance, which might be resulted from the electron transfer between Pd and the Cu(OH)_2_@CoNiCH/CF pre-catalyst [151]. For TMCHs-carbon material, Liu and co-workers prepared NiFeCH-carbon nanotubes and graphite (CNT/G) composite that NiFeCH embedded in carbon nanotubes and graphite [152]. The CNT/G in such compound enhanced the conductivity and combination between CNT/G and NiFeCH. Meanwhile, the special sandwich morphology (Fig. 6h) enabled more exposed active sites. All these merits caused improved OER performance, requiring 300 mV to deliver 10 mA cm^−2^ (Fig. 6i). As seen from these works, TMCHs-based heterostructures usually exhibit outstanding catalytic performance for OER. However, the structure evolution during OER becomes more complex due to multiple components, and therefore most works discuss the structure-performance relationship with the fresh heterostructure directly. In our view, the above-mentioned synergetic effects (e.g., electron transfer) should also exist in the after-reaction electrocatalysts, and researchers can probe them by using elaborate control experiments and thus provide a more convincing explanation.

### NO_3_^2−^

Although TMNHs also have abundant M–O bonds like TMCHs and thus are recognized as potential extraordinary pre-catalysts for OER, synthesis of TMNHs used to be a problem, as classical solvothermal methods have just been develop for TMNHs very recently [153].

**Fig. 7:**
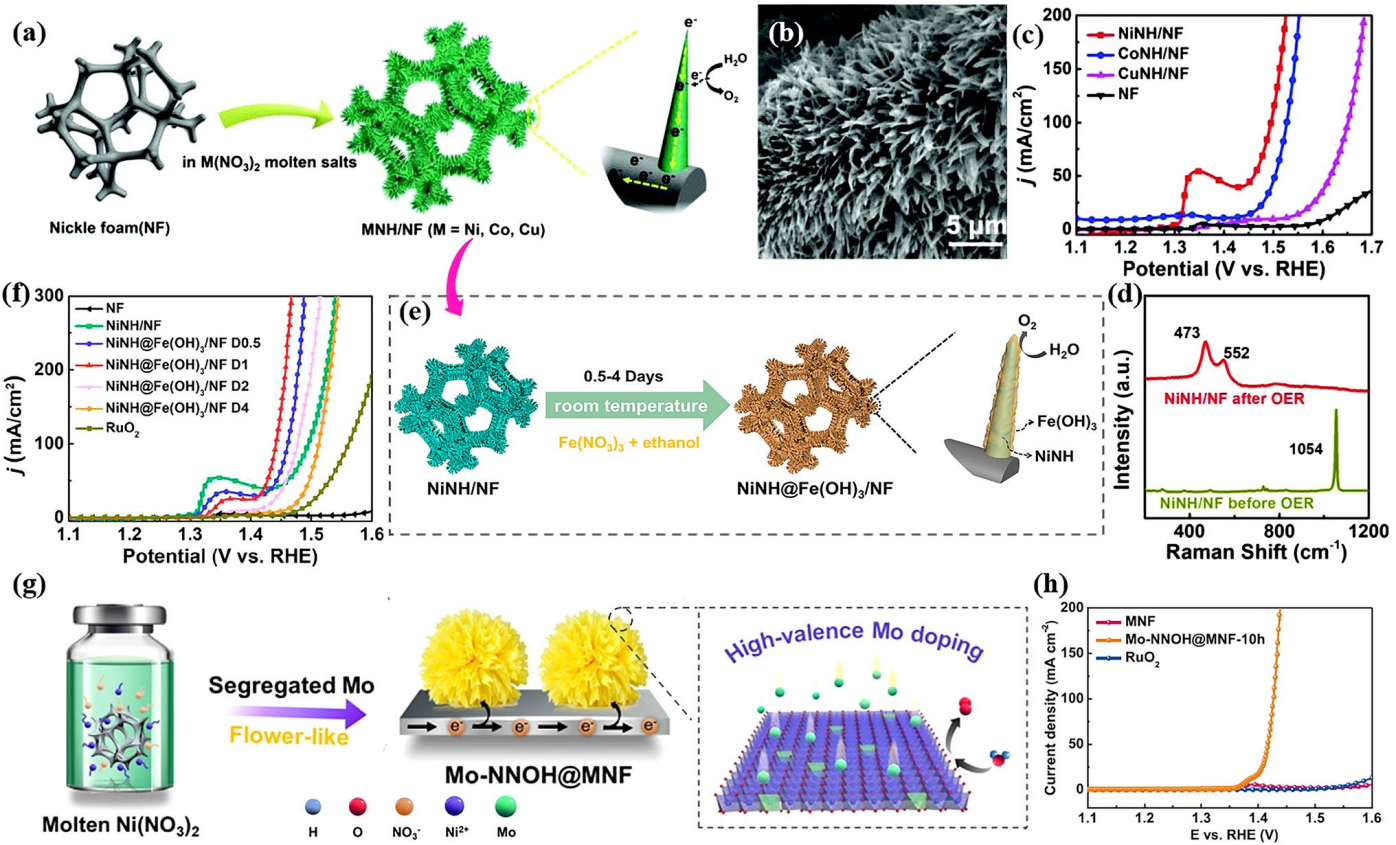
**a** Schematic illustration of the fabrication procedure of MNH/NF through molten salt decomposition method for OER. **b** SEM image of NiNH/NF. **c** LSV curves of MNH/NF samples and NF (after iR correction). **d** Raman spectra in comparison with NiNH/NF before and after OER. Reproduced with permission from Ref. [154]. Copyright 2018, Wiley–VCH Verlag GmbH & Co. KGaA, Weinheim. **e** Schematic illustration of the synthesis of NiNH@Fe(OH)_3_/NF by immersing NiNH/NF in Fe(NO_3_)_3_·9H_2_O ethanol solution. **f** OER electrocatalytic properties of NiNH@Fe(OH)_3_/NF materials in 1 M KOH solution. Reproduced with permission from Ref. [160]. Copyright 2020, American Chemical Society. **g** Schematic illustration of the fabrication procedure of Mo-NiNH on MNF substrate through molten salt decomposition strategy. **h** Polarization curves of Mo-NiNH @/MNF samples in 1 M KOH. Reproduced with permission from Ref. [161]. Copyright 2022, Elsevier Inc

To overcome this obstacle, our group came up with a novel molten salt decomposition method to fabricate multiple metal nitrate hydroxides (MNH, M = Ni, Co and Cu) on NF as OER pre-catalysts in alkaline media (Fig. 7a, b) [154]. This unique method utilizes the conversion of M(NO_3_)_2_ to MNH when the temperature reaches to the melting point, in which the molten nitrate is the only chemical regent involved in reaction process. The NiNH/NF showed the best OER performance among the three MNH/NF electrodes, which drove a catalytic current density of 50 mA cm^−2^ at an ultralow overpotential of 231 mV (Fig. 7c). After OER process, we found that the surface of NiNH and CoNH was oxidized to NiOOH and CoOOH, respectively, while the surface of CuNH was transferred to CuO. The generated metal oxyhydroxide or oxide was believed as true catalytically active species. Simultaneously, NO_3_^−^ could not be detected on the catalysts’ surface after carefully washing (Fig. 7d). However, recent report in this area indicated that the NO_3_^−^ would be dissolved into the electrolyte and adsorbed on the surface of MNH again, which was believed to facilitate the OER performance [155].

Considering the extraordinary OER performance of NiNH/NF, it is not surprised to have the follow-up works about metal doping and heterostructure. Fe- and Ni-based materials are well known as a great couple for OER [156–159]. Therefore, our group further fabricated amorphous Fe(OH)_3_ on NiNH/NF electrode to push forward its OER performance (Fig. 7e) [160]. As a result, the NiNH@Fe(OH)_3_ core@shell structure facilitated the charge transfer and built highly active Fe–Ni sites at the interface, leading to enhanced OER performance of 212 mV to drive a current density of 100 mA cm^−2^ (Fig. 7f). Chai et al. successfully prepared Mo-doped NiNH by simply replacing Ni foam with MoNi alloy foam (MNF) (Fig. 7g) [161]. The high valent Mo dopant effectively created defects and enlarged surface area, leading to high performance for OER (Fig. 7h). In our view, the NiMoNH/MNF electrode might possess great potential for the excellent performance for HER, because NiMo-based materials are almost the best non-noble metal-based HER catalysts to our best of knowledge. Dong et al. introduced B and Fe dopant into NiNH by submitting FeB supported on iron foam (IF) into molten nitrate [104], leading to a crystalline-amorphous structure and thus improved OER performance.

**Fig. 8:**
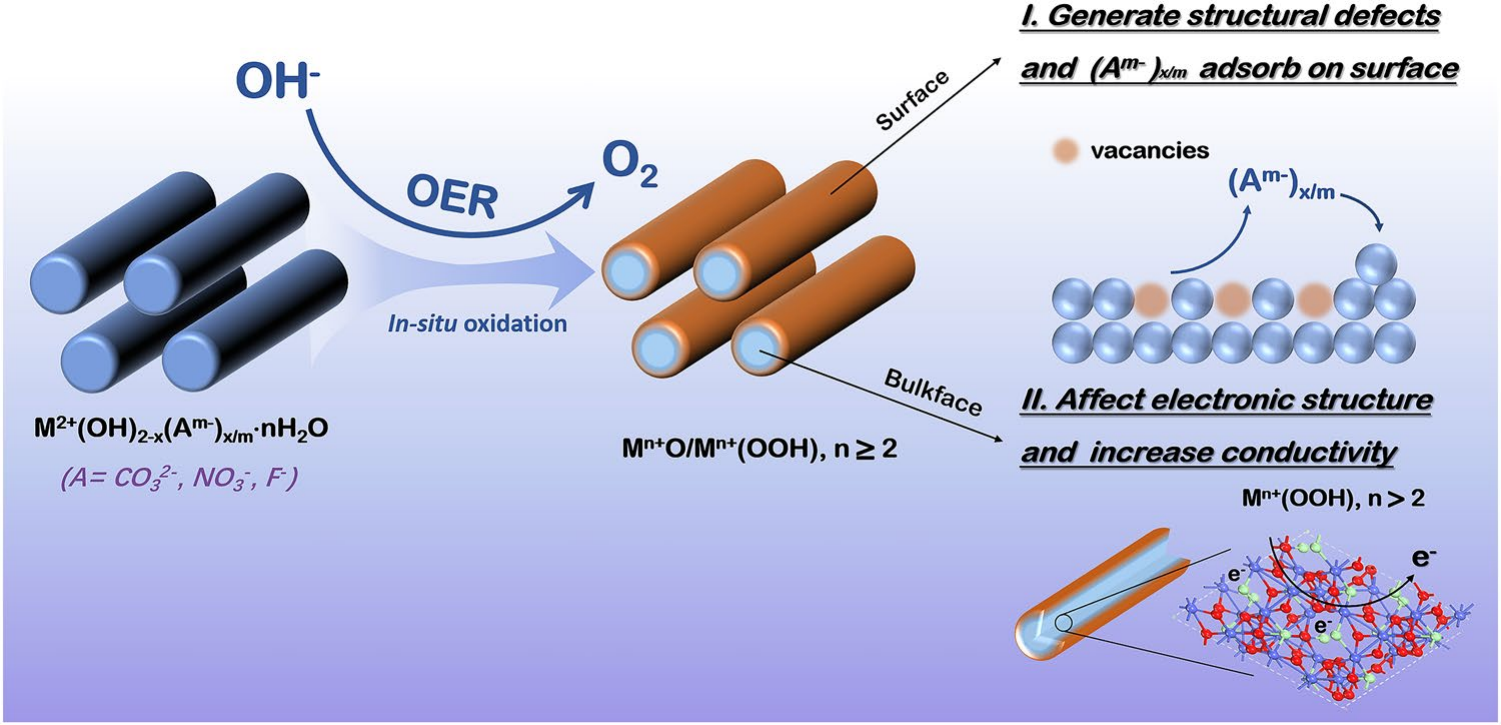
Schematic of structural evolution and anion effects for transition metal basic salts

As is discussed above, it is widely accepted that metal basic salts can be fully (CoClH) or partially converted to corresponding metal oxyhydroxides during OER, which is the real active center for OER. Understanding the impact of the anions (i.e. A^m−^) is drastically important for the rational design of the novel pre-catalysts. Taking the partially oxidized situation as an example, we have summarized the plausible anion effects for metal basic salts-based OER pre-catalysts in Fig. 8. On one hand, the oxidation of basic salts to MOOH is along with the leaching of A^m−^ to electrolyte, leaving the corresponding vacancies on the surface of catalysts. Simultaneously, the dissolved A^m−^ adsorbed on the surface of MOOH may influence on the adsorption of OER intermediate, which have been reported in metal sulfides and selenides pre-catalysts. On the other hand, metal basic salts in the core may not only provide higher conductivity than the hydroxide counterparts, but also affect the electronic structure of MOOH shell and further the OER performance. If theoretical calculations are employed to understand the anion effect, the factors of core–shell structure or vacancies or adsorption of anions should be considered for the corresponding effect.

## Transition Metal Basic Salts for Water Splitting

Compared to the single-function electrocatalysts, developing bifunctional catalysts that are simultaneously efficient for HER and OER remains challenging, but will be more appealing in practical applications. Transition metal basic salts are among the most promising non-noble metal-based pre-catalysts for OER, so it is of great value to investigate their catalytic activity towards HER to obtain catalysts for overall water splitting. Moreover, it is reasonable to speculate that metal basic salts can facilitate HER and further water dissociation in alkaline and medium solution due to their similar structure to metal hydroxides [149, 162].

To the best of our knowledge, there has no report about pure transition metal basic salts for HER and overall water splitting due to its poor instrinsic activity. However, modification strategies including but not limited to design of morphology/dimension [163, 164], modulation of electronic structure [147, 165], and interface engineering [95, 166, 167] have been introduced to improve the HER activity of metal basic salts. As a result, bimetallic and hetero-structured basic salts were employed as HER and further water splitting catalysts. In this part, recent advances of the modified transition metal basic salts toward these two topics are summarized and discussed in detail.

### Elemental Doping

**Fig. 9:**
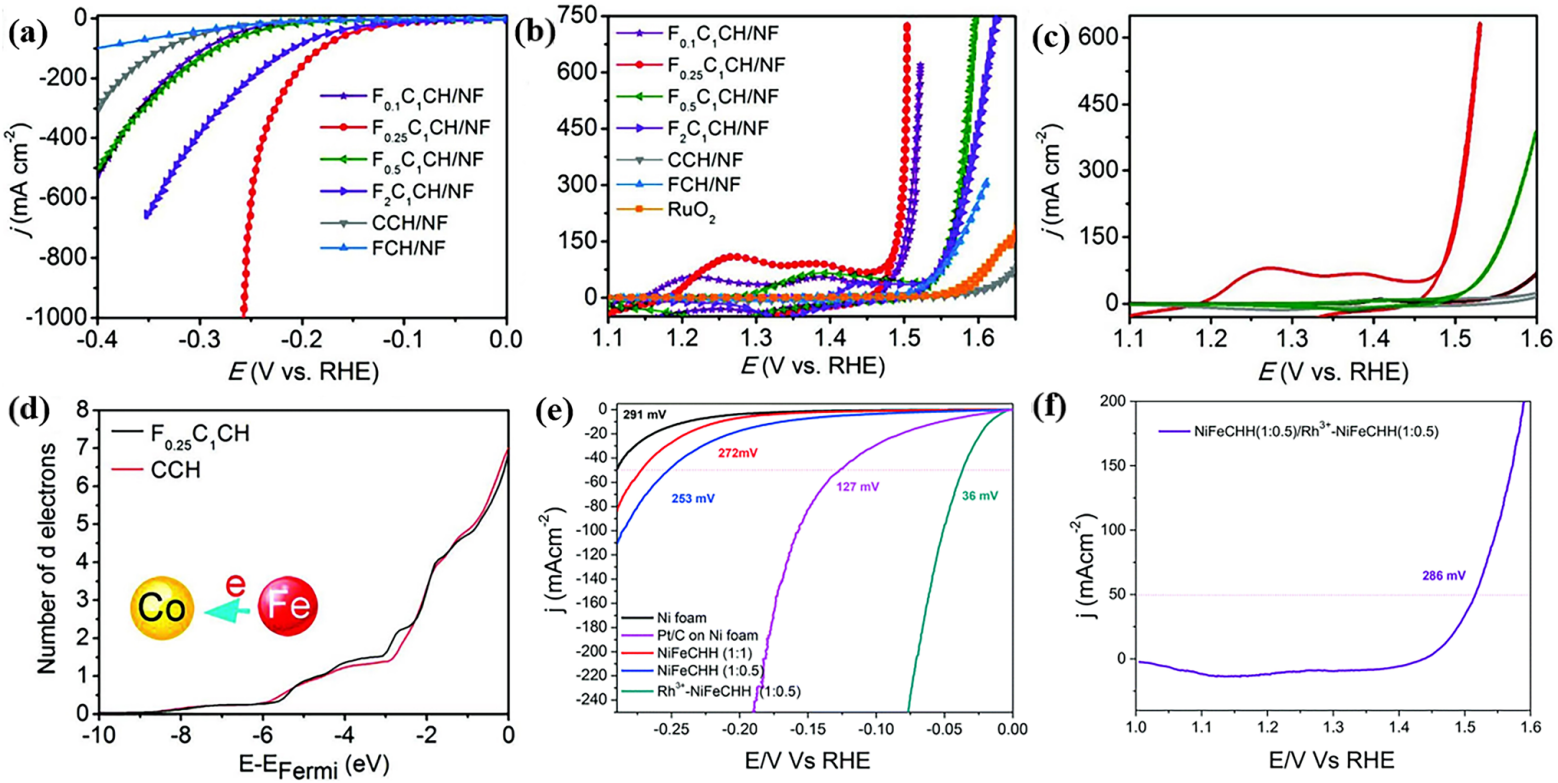
Polarization curves of various FeCoCH/NF samples for **a** HER, **b** OER and **c** overall water splitting. **d** The number of electrons in the 3d orbital per Co atom in CoCH and FeCoCH of Mo-NiNH @/MNF samples in 1 M KOH. Reproduced with permission from Ref. [118]. Copyright 2018, Wiley–VCH Verlag GmbH & Co. KGaA, Weinheim. Backward CV of sheet-like NiFeCH and Rh^3+^-NiFeCH toward **e** HER and **f** overall water splitting. Reproduced with permission from Ref. [169]. Copyright 2020, Royal Society of Chemistry

As mentioned in Sect. 3.3, Hu et al. first reported CoMnCH/NF for HER and overall water splitting. Although CoMnCH/NF with optimized Co:Mn ratio still required 180 mV to deliver a current density of 10 mA cm^−2^ for HER, which showed the possibility that TM basic salts can serve as catalyst for HER. Of note is that the catalyst exhibited good performance for overall water splitting (1.68 V @ 10 mA cm^−2^) thanks to its outstanding OER performance. In 2018, Li and his co-workers reported FeCoCH nanoarrays supported on NF as an excellent bifunctional catalyst for overall water splitting. The catalyst only needed 77 and 228 mV to deliver 10 mA cm^−2^ for HER and OER, respectively (Fig. 9a, b) [118]. Benefited from the high HER and OER activity, the electrolyzers only required a cell voltage of 1.45 V to drive identical current density (Fig. 9c), which is the best among non-noble metal-based catalysts for overall water splitting. After demonstrating the successfully doping of Fe into CoCH, the authors further found that the presence of strong coupling interactions and electron transfer between Fe, Co and O (Fig. 9d). By using density functional theory (DFT) calculations, the authors attributed the better HER performance of FeCoCH than CoCH to the moderated adsorption strength of H^+^ with catalyst surface. Moreover, W doped CoCH [147], and Co doped FeNiCH [168] have been reported as well due to the good performance for water splitting.

Of note is that though the HER performance of basic salts has been significantly enhanced through non-noble metal doping, there is still huge gap in performance compared to commercial Pt/C catalyst. Doping noble metal elements into transition metal basic salts can obtain overwhelming HER response. Kundu et al. have reported another Rh^3+^-NiFeCH for high-rate water electrolysis [169]. They claimed that the introduction of noble metal Rh^3+^ stabilized the NiFeCH systems and thus improved the inefficient HER kinetics, leading to a low overpotential of 36 mV to deliver a HER current density of 50 mA cm^−2^ (Fig. 9e). When employing NiFeCH as anode and Rh^3+^-NiFeCH as cathode, Rh^3+^ introduction easily induced the HER kinetics, decreasing the overall cell voltage remarkably. And hence, an overpotential of 286 mV was required to drive a current density of 50 mA cm^−2^ (Fig. 9f). Although noble metal doping can achieve excellent HER activity, it is essential to find a balance to combine noble metal and basic salts by considering the high cost of noble metal.

### Construction of Heterostructure/Composites

Besides for tuning the intrinsic HER activity of TM basic salts, another effective strategy is to build heterostructure of the TM basic salts and catalytic materials for HER and water splitting. On the one hand, a reasonable idea is to construct heterostructure of metal basic salts and typical HER-active materials such as Pt-group metal nanoparticles, metal sulfides, nitrides, carbides. On the other hand, some inert materials for HER may also be useful because they can tune the physical and chemical properties of basic salts to enhance their activity. Another question in this part is how to build the heterostructure of metal basic salts and the diverse HER active species, some of which containing low-valence metal species. Therefore, we not only discuss the relationship between structure-HER performance in detail, but also review the synthetic methods briefly.

**Fig. 10:**
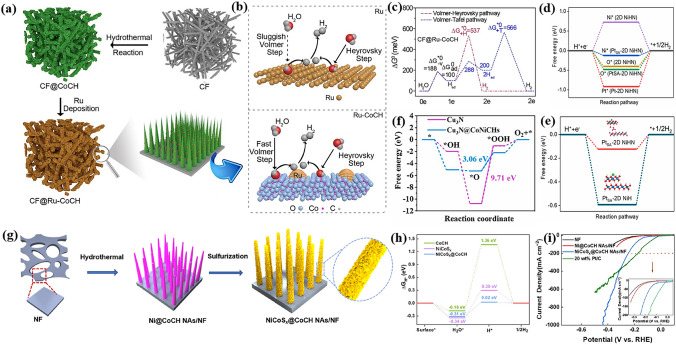
**a** Schematic illustration for the fabrication procedures and **b** synergistic effect of CF@Ru-CoCH samples. **c** Free energy diagram of the Volmer-Tafel and Volmer-Heyrovsky pathways for HER in alkaline electrolyte for CF@Ru-CoCH. Reproduced with permission from Ref. [170]. Copyright 2019, Elsevier Itd. **d** ΔGH* values at different sites on surface 2D NiHN and Pt_SA_-2D NiHN models towards HER at different sites on their surface. **e** ΔGH* comparison between Pt_SA_-2D NiHN and Pt_SA_-2D NiH models (inset: corresponding catalyst models). Reproduced with permission from Ref. [171]. Copyright 2022, Elsevier B.V. **f** Gibbs free energy diagram at 1.23 V for OER over Cu_3_N and Cu_3_N@CoNiCHs. Reproduced with permission from Ref. [173]. Copyright 2021, Elsevier B.V. **g** Schematic illustration of the fabrication procedure of NiCoS_x_@CoCH NAs/NF. **h** Calculated adsorption energies of H* and H_2_O* for CoCH, NiCoS_x_, and NiCoS_x_@CoCH. **i** Polarization curves of NF, Ni@CoCH NAs/NF, NiCoS_x_@ CoCH NAs/NF, and Pt/C (20 wt%). Reproduced with permission from Ref. [166]. Copyright 2021, American Chemical Society

Noble metal nanoparticles and metal basic salts should be a good couple for overall water splitting in alkaline solution, because it facilitates to the adsorption and desorption of atomic hydrogen and water dissociation. Ma and her co-workers deposited Ru on carbon fiber (CF)-supported CoCH to accelerate alkaline HER (Fig. 10a) [170]. The author showed that the CoCH reduced the energy barrier of water dissociation while Ru enabled to promote the formation of hydrogen gas (Fig. 10c). Thus, the synergistic effect between Ru nanoparticles and CoCH (Fig. 10b) improved the HER activity, requiring 66 mV to reach 10 mA cm^−2^. Very recently, Lee et al. fabricated Pt single atom (SA) on 2D NiHN via molten salt method followed by dipping the NiNH into 1 mM solution of H_2_PtCl_6_ [171]. The Pt_SA_-2D NiHN catalyst exhibited outstanding performance for HER (24 mV at 10 mA cm^−2^), OER (280 mV at 50 mA cm^−2^) and overall water splitting (1.45 V at 10 mA cm^−2^). The authors employed powerful theoretical calculations to explain the intrinsic HER activity of the Pt_SA_-2D NiHN catalyst. According to the calculated ΔG_H*_, the adsorption of H* on Ni sites in bare NiNH was too weak. Once Pt SA was added, the adsorption of H* on Ni sites is significantly strengthened, making the Ni sites in Pt_SA_-2D NiHN as the active sites for HER. Pt SA, known as robust active sites for HER, possessed too strong adsorption of H* in this work. Moreover, the authors further investigated the anion effect for HER. They discovered that the well achieved ΔG_H*_ closer to 0 for the Pt_SA_-2D NiHN was also correlated to the NO_3_^−^, because the ΔG_H*_ for Pt SA/NiOH (marked as NiH in this work) is much lower, indicating an extremely strong of H* intermediate. The NO_3_^−^ in Pt_SA_-2D NiHN could adjust the bonding electron density and further the adsorption of H* and finally the HER performance. In our view, this work is a milestone for metal basic salts-based catalysts for overall water splitting, because the authors not only report a catalyst with outstanding catalytic performance for HER and further overall water splitting, but also well establish the structure-performance relationship through powerful theoretical calculations and successfully guide the design of future electrocatalysts. Besides, FeNi alloy quantum dot@CoCH/NF has also been reported [172]. Considering the high-price of noble metal-based materials, it is cost-effective to combine TM basic salts and non-noble metal-based catalysts for HER. For example, Luo and co-workers synthesized a 3D hierarchical hetero-structured Cu_3_N@CoNiCHs@copper foam (CF) electrode material through hydrothermal method to be efficient catalyst for overall water splitting [173]. The interaction between Cu_3_N (with better HER activity) and CoNiCH generated electron deficient Co sites in CoNiCH and electron rich Cu sites in Cu_3_N (Fig. 10f), which were favorable to synergistically enhance the OER and HER activity, respectively. Given this reason, the Cu_3_N@CoNiCHs@CF electrode required a low cell voltage of 1.58 V to reach 10 mA cm^−2^. Besides, Song and co-workers reported a hetero-structured NiCoS_x_@CoCH catalyst through a process of sulfurization at room temperature (Fig. 10g) [166]. The obtained NiCoS_x_/CoCH/NF electrode displayed excellent HER performance due to the improved water adsorption and splitting kinetics (Fig. 10h, i). Furthermore, sulfured NiCoCH/NF [163], Se nanoparticles modified CoCH with in-situ generated Co-Se [174], and graphdiyne (GDY) modified FeCH [165] have also shown superior HER activity because of the introduction of proper components. However, the overall water splitting performance of these catalysts is not measured. Considering the enhanced HER activity and the intrinsically good OER activity of TM basic salts, we believe that these materials listed above can have promising activity toward overall water splitting.

**Fig. 11:**
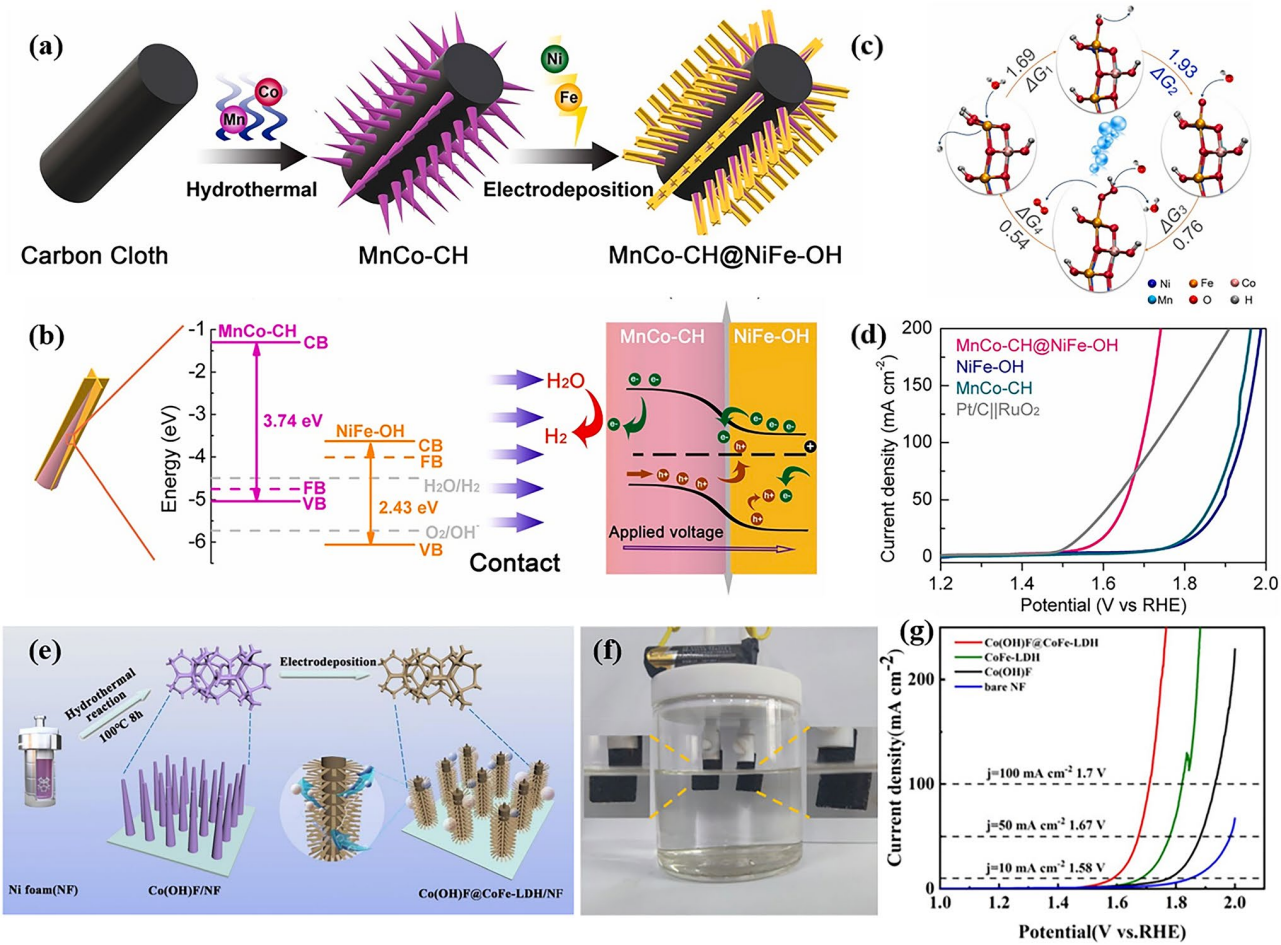
**a** Schematic illustration for the synthetic process of MnCo-CH@NiFe-OH pn junction. **b** Energy diagrams of the MnCo-CH and NiFe-OH (left) and the MnCo-CH@NiFe-OH pn junction (right). **c** Free energetic pathway of water oxidation over MnCo-CH@NiFe-OH. **d** IR-corrected LSV curves of MnCo-CH@NiFe-OH toward overall water splitting. Reproduced with permission from Ref. [175]. Copyright 2021, Elsevier B.V. **e** Schematic illustration for the step-wise fabrication of the Co(OH)F@CoFe-LDH heterostructure supported on NF. **f** Digital photograph of the setup for water splitting driven by one battery. **g** Polarization curves of various Co(OH)F@CoFe-LDH sample. Reproduced with permission from Ref. [95]. Copyright 2022, Royal Society of Chemistry

Inert materials for HER, such as metal oxides and hydroxides, can behave as efficient promoters to enhance catalytic activity toward OER and overall water splitting. For instance, Wang and his co-workers demonstrated a hierarchical core–shell structured MnCo-CH@NiFe-OH *pn* junction catalyst by electrodeposited method and employed it to overall water splitting (Fig. 11a) [175]. It is demonstrated that the contract of HER active MnCo-CH and OER active NiFe-OH led to a band bending, which widened the gap between the valence band of MnCo-CH and theoretical HER potential, significantly promoting electron transfer for generating H_2_. Meanwhile, the positively charged NiFe-OH was considered to facilitate the transfer and adsorption of OH^−^ to boost OER activity (Fig. 11b). DFT results further indicated that the desorption of H atom from adsorbed OH* (rate-limiting step, ΔG_2_) can be influenced by the formation of pn junction, which made it easier to release H atom (Fig. 11c). Therefore, with both enhanced HER and OER activity, a low cell voltage of 1.69 V was obtained to drive a current density of 100 mA cm^−2^ (Fig. 11d). Moreover, Wang et al. fabricated hetero-structured Co(OH)F@CoFe-LDH/NF catalyst via a facile two-step approach and used to overall water splitting (Fig. 11e, f) [95]. The author declared that the synergistic effect at the interface that generated by covering CoFe-LDH nanosheets on Co(OH)F nanorod was favorable to catalytic activity. Thus, the Co(OH)F@CoFe-LDH/NF electrode displayed superior electrocatalytic activity, achieving a current density of 10 mA cm^−2^ at a small cell voltage of 1.58 V (Fig. 11g). Besides, TiO_2_@CoCH [164], and CoCH@FeOH [176] were also reported with enhanced performance of HER or overall water splitting.

## Conclusion and Perspective

Development of highly efficient non-noble metal based electrocatalysts towards OER, which is the bottleneck of H_2_ production from overall water splitting, has long been an important topic in the field of electrocatalysis. In the past ten years, transition metal basic salts with F^−^, Cl^−^, CO_3_^2−^ and NO_3_^−^ have attracted extensive research interest because they exhibit much better OER activity than the conventional used metal hydroxide-based materials OER due to the anions. Despite the complexity originated from the structure evolution of basic salts during OER, some self-consistent models including anion defect model and core–shell model have been established for understanding the effect of anions on the final OER performance. Moreover, strategies such as metal doping and construction of heterostructure have been employed not only to push forward the OER performance, but also endow basic salts the ability to catalyze HER and overall water splitting. Despite the great progress that has been made, there are still some challenges and opportunities for this emerging catalyst.

### Understanding the Effect of Anions

The anions contained in transition metal basic salts play a crucial role in the outstanding OER performance of metal basic salts. It is crucial to address issue for rational design of pre-catalysts. Till now, most of the works explain this important issue by simply carrying out DFT calculations with basic salts as models. However, all kinds of metal basic salts undergo partially or even fully oxidation during OER test, which means that most present explanations are not appropriate. Although the self-consistent defect models and core–shell models have been raised for explaining the effect of Cl^−^ and F^−^, respectively, there has not been a general model for all the kinds of basic salts. In other words, a systematic research work which involves all the present metal basic salts with F^−^, Cl^−^, CO_3_^2−^ and NO_3_^−^ is needed to give a reasonable explanation for the effect of anions. Moreover, considering the complexity of anion effect, *in-situ* and *operando* means such as XAFS, XPS, Raman spectroscopy should be utilized to give deep insight to metal basic salts OER pre-catalysts.

### Development of New Transition Metal Basic Salt-based Pre-catalysts

Heretofore, there are four kinds of metal basic salts in terms of acid group anion (F^−^, Cl^−^, CO_3_^2−^ and NO_3_^−^) have been developed as catalysts towards OER, HER and overall water splitting. Plenty of follow-up works have been reported to improve the catalytic performance of these basic salts, however, metal basic salts with other anions (e.g., SeO_4_^2−^, PO_4_^3−^, CH_3_COO^−^) have not been employed as catalysts. In our view, these metal basic salts should also be promising pre-catalysts towards OER. Therefore, it is a research opportunity to develop new kind of metal basic salts as pre-catalyst towards OER.

### Practical Application of Metal Basic Salt-based Pre-catalysts

The large current density and stability of OER electrodes are critical to the practical application of water splitting catalysts. As far we know, some of transition metal basic salts, such as MCH, have shown promising catalytic activity under industrial-level current density (1000 mA cm^−2^). However, the catalytic performance of most transition metal basic salt-based pre-catalysts is still evaluated at low OER current density (≤ 100 mA cm^−2^). Thus, it is encouraged to explore the catalytic performance of transition metal basic salt-based pre-catalysts at industrial-level current density to promote its practical application.

### Exploration of New Anodic Reaction

Considering the outstanding OER activity of metal basic salts, it is reasonable to use these pre-catalysts in another electrochemical oxidation reaction. For example, urea oxidation reaction (UOR) is regard as the more favorable anodic reaction due to the lower cell potential of 0.37 V (vs. RHE), which is much lower than that of OER (1.23 V vs. RHE). Up to date, there are only a few reports in this topic with TMCH as the pre-catalyst [177], so some interesting works are expected in this area.
